# Diagnosis of Fanconi Anemia: Mutation Analysis by Next-Generation Sequencing

**DOI:** 10.1155/2012/132856

**Published:** 2012-06-03

**Authors:** Najim Ameziane, Daoud Sie, Stefan Dentro, Yavuz Ariyurek, Lianne Kerkhoven, Hans Joenje, Josephine C. Dorsman, Bauke Ylstra, Johan J. P. Gille, Erik A. Sistermans, Johan P. de Winter

**Affiliations:** ^1^Department of Clinical Genetics, VU University Medical Center, Van der Boechorststraat 7, 1081 BT Amsterdam, The Netherlands; ^2^Department of Pathology, VU University Medical Center, De Boelelaan 1117, 1081 HV Amsterdam, The Netherlands; ^3^Leiden Genome Technology Center, Center for Human and Clinical Genetics, Leiden University Medical Center, Einthovenweg 20, 2333 ZC Leiden, The Netherlands

## Abstract

Fanconi anemia (FA) is a rare genetic instability syndrome characterized by developmental defects, bone marrow failure, and a high cancer risk. Fifteen genetic subtypes have been distinguished. The majority of patients (*≈*85%) belong to the subtypes A (*≈*60%), C (*≈*15%) or G (*≈*10%), while a minority (*≈*15%) is distributed over the remaining 12 subtypes. All subtypes seem to fit within the “classical” FA phenotype, except for D1 and N patients, who have more severe clinical symptoms. Since FA patients need special clinical management, the diagnosis should be firmly established, to exclude conditions with overlapping phenotypes. A valid FA diagnosis requires the detection of pathogenic mutations in a FA gene and/or a positive result from a chromosomal breakage test. Identification of the pathogenic mutations is also important for adequate genetic counselling and to facilitate prenatal or preimplantation genetic diagnosis. Here we describe and validate a comprehensive protocol for the molecular diagnosis of FA, based on massively parallel sequencing. We used this approach to identify *BRCA2*, *FANCD2*, *FANCI* and *FANCL* mutations in novel unclassified FA patients.

## 1. Introduction

Fanconi anemia (FA) is a recessive chromosomal instability syndrome with diverse clinical symptoms and a high risk for acute myeloid leukemia and squamous cell carcinoma of the head and neck region [1]. Clinical suspicion of FA is mostly based on growth retardation and congenital defects in combination with life-threatening bone marrow failure (thrombocytopenia and later pancytopenia), which usually starts between 5 and 10 years of age. However, the clinical manifestations of FA patients are highly variable, and therefore the FA diagnosis should be confirmed by a positive chromosomal breakage test and/or pathogenic mutations in one of the FA genes. Currently, mutations in 15 different genes are known to cause FA, and their gene products act in a pathway that takes care of specific problems that may arise during the process of DNA replication [2].

The conventional Sanger sequencing-based mutation screening approach for FA is time-consuming, costly, and most importantly may not detect all types of disease-causing aberrations, such as deep intronic mutations, large deletions, and amplifications. Furthermore, the existence of *FANCD2 *pseudogenes obstructs the identification of pathogenic mutations in this gene when sequencing genomic DNA. Here, we demonstrate a comprehensive mutation detection approach for FA based on massively parallel sequencing (MPS) [3].

## 2. Methods

We designed an in-solution oligonucleotide hybridization capture kit (SureSelect, Agilent) targeting the open reading frames of all FA genes, except for regions that contain repetitive and low complexity DNA sequences as assessed by RepeatMasker (http://www.repeatmasker.org/). All exons-, 3′-, and 5′-UTR-regions, and exon-intron boundaries were targeted by this approach (the oligonucleotide locations, in a  .BED format, are available upon request). In addition, a number of other genes involved in cancer predisposition and routinely screened in our diagnostic lab were included in the enrichment kit. We used the Illumina GAIIx platform for sequencing.

To assess the performance of the custom target kit and the massively parallel sequencing method, we selected FA samples with a spectrum of different types of known variations (Table 1). The pathogenic mutations in these samples have previously been identified either by Sanger sequencing or by multiplex ligation-dependent probe amplification (MLPA) [4]. One of the samples included in the study was from a carrier of a *BRCA1* mutation. Unique barcode sequences were used for 11 DNA libraries to allow distinction between the samples that were run on a single Illumina flow-cell lane. In addition, to evaluate the sensitivity of the approach, two DNA samples were pooled before library preparation to mimic a mosaic condition.

An in-house variation detection pipeline, including a novel tool for large deletion detection, was used to score for relevant mutations.

### 2.1. Library Preparation

For each sample, 1.5 *μ*g of DNA was resuspended in 75 *μ*L of TE buffer in a Covaris microTube, and subsequently sheared in a Covaris S220 (Covaris, Inc. MS, USA) using the following settings: duty cycle = 10%, Intensity = 5, cycles per burst = 200, time = 360, Set mode = frequency sweeping, and temperature = 4°C. Fragmented DNA containing overhangs is converted into blunt ends using T4 DNA polymerase and Klenow enzyme (New England Biolabs) by incubation for 30 minutes at 20°C. The DNA sample is then purified with QIAquick PCR purification kit (Qiagen) following the manufacturer's instructions and eluted in 32 *μ*L of Qiagen elution buffer. Next, the 3′ ends of the fragmented DNA are adenylated using Klenow exonuclease (New England Biolabs) by incubating for 30 minutes at 37°C. The DNA sample is then purified with MinElute PCR purification kit (Qiagen) following the manufacturer's instructions and eluted in 10 *μ*L of Qiagen elution buffer. Next, Illumina-specific index paired-end adapters (Illumina) are ligated to the 5′ and 3′ ends of DNA fragments by incubation with DNA ligase (New England Biolabs) for 15 minutes at 20°C. The adapter-ligated DNA fragments are purified with MinElute PCR purification kit, and 1 *μ*L is used to assess proper adapter ligation by a control PCR using InPE 1.0, InPE 2.0, and a random index primer with the following PCR conditions: 30 sec at 98°C, 18 cycles of 10 sec at 98°C, 30 sec at 65°C, 30 sec at 72°C, and a final step of 5 minutes at 72°C. The quality and quantity of the library is evaluated with the Agilent 2100 Bioanalyzer on a DNA 1000 chip following the manufacturer's instructions.

For the FA gene enrichment, we used 500 ng of adapter-ligated library following the manufacturer's instructions. Briefly, DNA libraries are incubated with the biotinylated RNA custom SureSelect library “baits” for 16 hours at 60°C. Next, DNA library that hybridized to the baits is captured using magnetic beads (Dynabeads, Invitrogen), washed, and eluted in elution buffer. Primers containing unique barcode sequences are used to amplify captured libraries, and equimolar pooling is performed after quantification on a bioanalyzer. The eleven pooled DNA libraries were then sequenced on a single flow cell lane of an Illumina GAIIx using a 72 cycle multiplex paired-end sequence protocol.

## 3. Results

**Figure 1:**
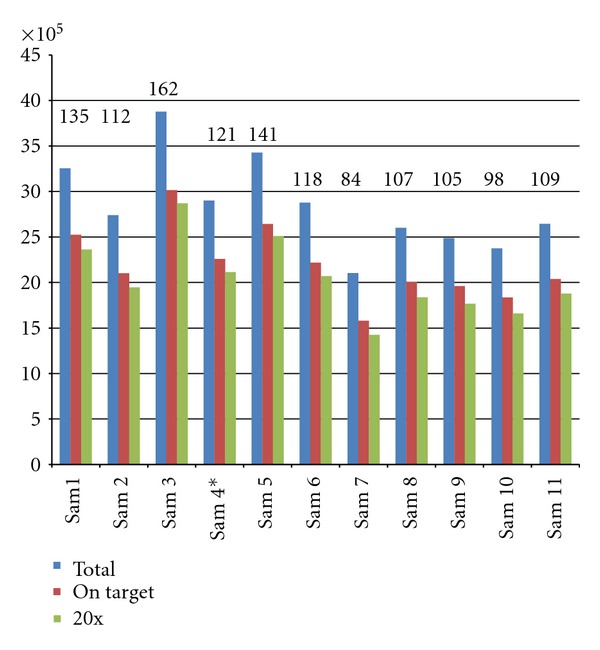
Specifications of 11 DNA libraries pooled and sequenced on one flow cell lane of an Illumina GAIIx. Numbers on top of the bars indicate the average sequencing depth obtained for the individual samples. Blue bars indicate total number of unique reads, red bars indicate reads that fall on target of the bait design, green bars indicate reads that fall in target regions covered with at least 20x depth. Sample 4 is a mix of genomic DNA from two individuals 4a and 4b.

Sequence data from DNA libraries of eleven carriers of mutations in the FA genes: *FANCA (4), FANCB (1), FANCC (1), FANCD1 (1), FANCE (1), FANCG (1), FANCI (1), FANCN (1)*, and one individual carrying a mutation in *BRCA1,* were generated from an Illumina GAIIx sequencer. An average of 2.8 million unique reads were obtained per library resulting in a median sequence depth of about 100 fold, with an average enrichment efficiency of >75% (Figure 1). Several types of disease-causing genetic aberrations were present in the assayed DNA samples including single nucleotide substitutions, small deletions (1–8 nucleotides), and large deletions (multiple exons). We developed a variation detection pipeline detecting all these types of aberrations.

### 3.1. Detection of Single Nucleotide Variations (SNVs) and Small Insertions/Deletions (Indels)

The data analysis pipeline for the detection of SNVs and small indels that was developed is comprised of freely available tools on the web. An initial quality check of the sequence reads is followed by mapping the paired-end reads with the Burrows-Wheeler Aligner (BWA) [14] to the National Center for Biotechnology Information (NCBI) hg19 build reference genome. Subsequently, SNPs and small indels are called using Samtools [15] and Varscan [16]. The resulting list of variations is annotated with Annovar [17] that utilizes information from external databases to generate context around the mutations, such as amino acid change consequence, location to canonical splice site regions, and information about reference to dbSNP and frequencies if available. Finally, a manual filtering step is executed to prioritize relevant mutations. Low-frequency frameshifts and truncating mutations are considered pathogenic. Unreported nonsynonymous amino acid variations are analyzed *in silico* by the pathogenicity predicting programs, Align-GVGD, Polyphen-2 and SIFT [7–9] to help assess the damaging effect. 

The variation detection frequency for all samples was set at a minimum of 31% of the reads covering the aberrations except for sample 4 (pre-library DNA pooling of samples 4a and 4b), for which it was set at 12%. The total number of variations detected in the samples and the subsequent reduction in number of variations through filtering is depicted in Table 2.

Using the variation detection pipeline and filtering procedure 12 SNVs out of the total of 13 were detected. The variation that escaped the initial filtering procedure resided deep in the intronic region; 920 nucleotide upstream of the exon start site. However, the variation was present in the initial variation list.

### 3.2. Detection of Large Insertions/Deletions

Large deletions are detected by analyzing local read depth. Firstly, a reference local read depth is established by binning read counts in a preset sliding window using data from all pooled samples. The local read depth is also determined for each sample separately, using the same preset sliding window used for the reference. A Log2 ratio is calculated for each window by dividing the local read depth of the sample by the reference. Normalization is performed through a mean shift to zero. The copy number data is projected on the open reading frame (ORF) of the gene and also projected on an exon scale, where the mean read count is aggregated on a per exon basis.

**Figure 2:**
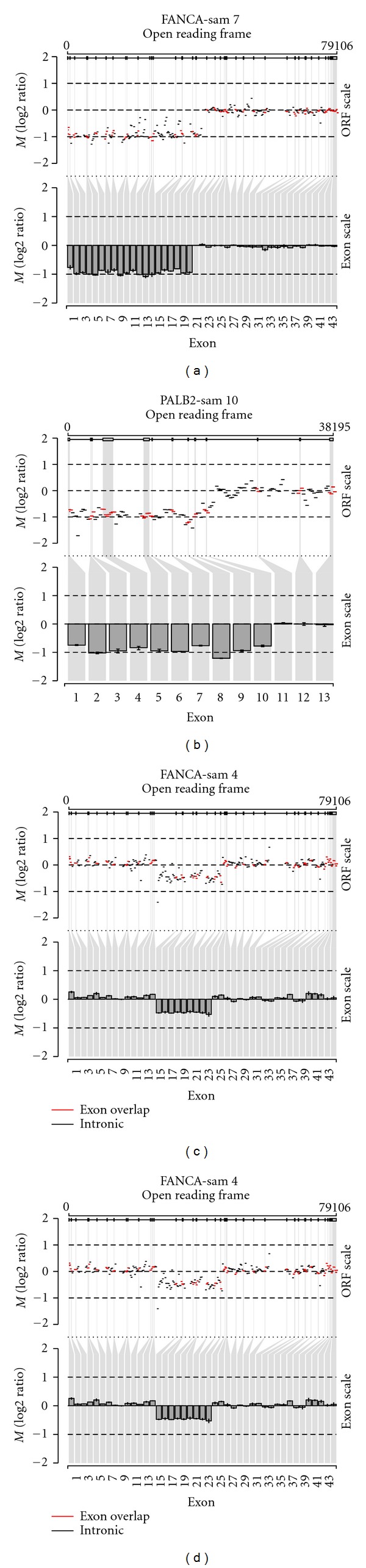
Large detection using next-generation sequence data. Copy number data are projected on an open reading frame (ORF) and on an exon scale. The ORF scale (upper panel) shows log2 ratios (*M*) for all exons and introns. Red segments indicate an overlap with an exon and black segments indicate no overlap with exons. The exon scale (lower panel) only shows the mean log2 ratio per exon with their 25th and 75th percentile. *R* plots of large deletion analysis. Deletion of exons 1 to 20 of *FANCA* (a), exons 1 to 10 of *FANCN* (b), exons 15 to 23 in *FANCA* (c), and exon 38 in *FANCI* (d).

**Figure 3:**
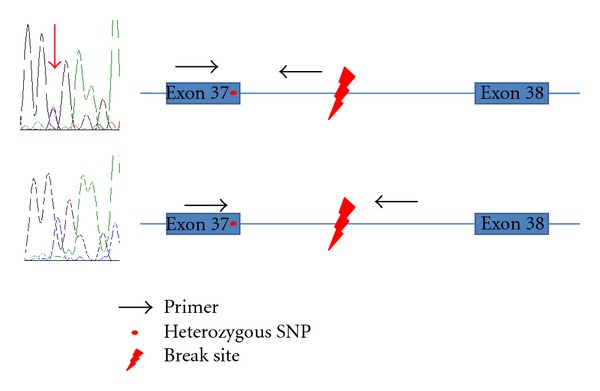
Confirmation of the large *FANCI* deletion by Sanger sequencing. A part of exon 37 with a SNP was amplified with primers designed either up- or downstream of the deletion breakpoint. Sequence analyses resulted in the detection of the SNP (red arrow) as heterozygous or hemizygous, respectively.

The large *FANCA* and *PALB2* deletions, previously identified by MLPA (Table 1), were readily detected using the MPS data. The DNA from sample 4b that contained a heterozygous exon 15 to 23 deletion in *FANCA* was pooled with sample 4a before library preparation. As expected, the read depth Log2 ratio as compared to the reference was approximately −0.5, which corresponds with a loss of one allele in a background of four copies. Interestingly, in sample 11 only one pathogenic nucleotide substitution in *FANCI* has previously been identified while the other mutation remained undetected. Here we show the deletion of the last exon (exon 38) of the gene as detected by our large indel analysis tool (Figure 2(d)). We confirmed the deletion by PCR and Sanger sequencing by using a SNP in the last unaffected exon 37 and two sets of primers amplifying up- and downstream of the breakpoint (Figure 3).

### 3.3. Identification of Pathogenic Mutations in Unclassified FA Patients

To investigate if our next-generation sequencing method also identifies mutations in unclassified FA patients, we investigated five patients that were sent in for FA mutation screening, without prior knowledge about their gene defect. Aberrations in the *FANC-A*, -*C*, -*E*, -*F*, and -*G *genes were already excluded in a routine diagnostics analysis by MLPA and Sanger sequencing. In two patients we identified known compound heterozygous pathogenic mutations in *BRCA2* and *FANCD2*. In two other patients, novel homozygous mutations were detected. In one patient (Unc4) a dinucleotide insertion/duplication in exon 9 of *FANCL* was found, which results in a frameshift and a stop codon in exon 14. The other patient (Unc3) showed a missense mutation in exon 28 of *FANCI* that changed codon 954 from a cysteine to a tyrosine. The affected amino acid is highly conserved, up to fruit fly, suggesting that it may have an important function. Moreover, *in silico* analysis by SIFT [9] and Polyphen 2 [8] predicted the amino acid change to be damaging. All the identified variations were confirmed by Sanger sequencing, and the novel variations had a proper segregation within the family.

In one patient no pathogenic variants were detected, although the coding regions of all known FA genes were covered deep enough to call variants. This suggests that the patient has a defect in a new FA gene or is not a true FA patient.

## 4. Discussion

The mutation detection strategy described here proved to be efficient for the molecular diagnosis of FA although patients with mutations in *FANCF*, *-J*, *-M*, *-O,* and -P were not included in our study. All the FA mutations identified by Sanger sequencing were also detected by next-generation sequencing. Moreover, we discovered a novel large deletion in the FA-I patient, for whom only one truncating mutation was previously identified [4]. The exact breakpoint at nucleotide level could be distinguished as also the intronic regions of the FA genes were enriched and sequenced. We confirmed the deletion by PCR and Sanger sequencing, using a SNP in the last unaffected exon and two sets of primers amplifying the regions up- and downstream of the breakpoint (Figure 3). Besides this novel large deletion we identified large genomic deletions in *FANCA* (2 samples) and *PALB2* (1 sample). The sensitivity of the method was demonstrated by mixing two DNA samples prior to library preparation and enrichment. A deletion of one allele in a background of four alleles can be detected, suggesting that the method is even applicable for the classification of mosaic FA patients. However, a thorough assessment of the method using serial dilutions with samples harboring large deletions is required to determine the detection limit of the assay. The identification of large deletions is essential for FA molecular diagnostics since about 40% of pathogenic mutations in the major FA complementation group, FA-A, are caused by large deletions in *FANCA*. As large deletions have also been demonstrated for *FANCI* (this study) and *FANCN *[6], it is plausible that these types of aberrations are present in other FA samples, which were previously unclassified by conventional molecular screening methods. Therefore, these types of mutations should be examined in the standard molecular diagnostics of FA.

The presence of *FANCD2* pseudogenes can complicate the identification of pathogenic mutations in this gene, as variations will tend to have a reduced frequency due to the occurrence of multiple highly similar copies. However, this difficulty can be resolved with bioinformatics by flagging variations that tend to have lower frequencies than expected within those regions. In cases where all other FA genes are excluded for mutations, careful inspection is required for flagged variations. Deep intronic mutations represent another type of variation, which are not analyzed with the classical molecular diagnostics approach. Indeed, the hemizygous *FANCA* c.893+920 C>A mutation in sample 7 was only identified after a heterozygous large *FANCA* deletion was detected by MLPA. This suspected the presence of a mutation on the other *FANCA* allele, which was then found after the FANCA cDNA was analyzed [5]. 

When we applied our novel molecular diagnostics approach on unclassified FA patients, we identified pathogenic mutations in four individuals. All the variations were confirmed with Sanger sequencing and demonstrated proper segregation with the disease. Two patients carried biallelic mutation in *BRCA2* and *FANCD2*, which have been described previously (Table 3). The remaining two patients harbored novel homozygous mutations in *FANCI* and *FANCL*; c.G2861A (p.C954Y) and c.755_756insAT (p.M252fs), respectively. The frameshift mutation in *FANCL* results in a stop codon in the last exon of the gene, which likely produces an mRNA targeted for nonsense mediated decay. The c.2861G>A substitution in *FANCI* affects a highly conserved amino acid and results in a large physiochemical difference, which is predicted to have a damaging effect on the FANCI protein by SIFT and polyphen-2. Nevertheless, functional studies are required to ultimately classify this variation as pathogenic. In one FA sample we could not detect the disease causing mutations in any of the known FA genes. This patient might represent a novel FA subtype and whole-exome sequencing might be a useful approach to identify the affected gene.

**Figure 4:**
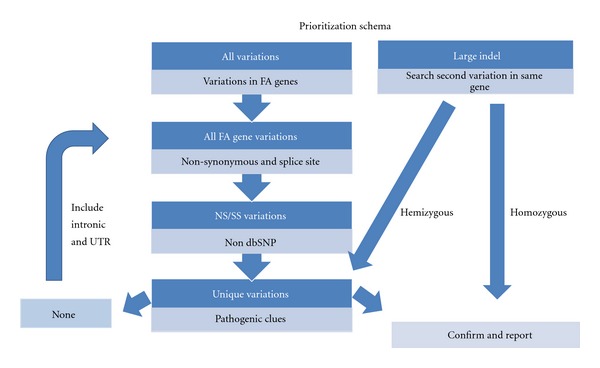
Prioritization scheme for the detection of disease-causing mutations by next-generation sequencing.

Altogether, inspection of different variation types and inclusion of intronic regions warrants a comprehensive molecular FA diagnosis. Given the average number of variations of around 2500 per patient, it appears a difficult task to recognize the disease-causing mutation(s). Here we propose a prioritization approach following the recessive mode of inheritance (Figure 4). When no large deletions are identified, an initial filtering for nonsynonymous and canonical splice site variations should be performed. Subsequently, exclusion of variations in dbSNP and an in-house variant database with a frequency above 2%, reduces the number of possible pathogenic variations to less than 4. In cases where only one heterozygous pathogenic mutation is found, close examination of variations in intronic- and UTR regions in the same gene is required. When no pathogenic variations have been detected, assessment of all unique intronic and UTR variations is needed. With an ever expanding variant database of characterized FA patients, the identification of pathogenic mutations will become less complicated. Nevertheless, the necessity for functional tests, such as retroviral complementation or transfection, will remain essential to help assess the pathogenic status of unclassified missense variants.

In conclusion, multiplexed next-generation sequencing based on massively parallel sequencing is an effective molecular diagnostics approach for FA. The procedure, performed on genomic DNA, reduces the turnaround time, number of assays, and costs for a reliable detection of the disease-causing mutation. With the ever decreasing costs of enrichment and sequencing procedures, we expect that in the near future this will be the first test for patients clinically suspected of FA, thus avoiding labor-intensive chromosomal breakage assays and reducing turnaround time for FA diagnosis. To increase the efficiency of the molecular diagnosis, genes involved in other bone marrow failure syndromes (e.g., diamond-Blackfan anaemia and Shwachman-diamond syndrome) can be included, to be able to diagnose these non-FA patients that are often referred for FA diagnosis.

## Figures and Tables

**Table 1:** Selected FA samples with mutations previously identified by Sanger sequencing and multiplex ligation-dependent probe amplification (MLPA), used for validation of the next-generation sequencing approach.

Sample (affected gene)	Allele 1	Allele 2	Reference
Sam 1 (*BRCA2*)	c.9253dupA	—	BIC DB#
Sam 2 (*FANCA*)	c.3558insG	—	[4]
Sam 3 (*FANCG*)	c.271–272del	c.620delT	[4]
Sam 4a (*FANCA*)	c.1464C>G	c.2632G>C	[4]
Sam 4b (*FANCA*)	Ex 15–23 del	—	[4]
Sam 5 (*BRCA1*)	c.2694dupA	—	BIC DB#
Sam 6 (*FANCC*)	c.376–377del	c.844-1G>C	[4]
Sam 7 (*FANCA*)	Ex 1–20 del	c.893+920 C>A	[5]
Sam 8 (*FANCB*)	c.811insT	absent	[4]
Sam 9 (*FANCE*)	c.91C>T	c.91C>T	[4]
Sam 10 (*PALB2*)	Ex 1–10 del	c.1802T>A	[6]
Sam 11 (*FANCI*)	c.2509G>T	NF*	[4]

#Breast cancer information core database available at http://www.research.nhgri.nih.gov/bic/.

*NF: not found.

**Table 2:** Pathogenic mutation detection through filtering.

Sample^1^	Variants (total)	Variants (target genes)	NS/SS^2^	Not in dbSNP	FA genes^3^	Pathogenic clue^4^
Sam 1	2535	1579	58	11	4	1
Sam 2	2659	1700	43	6	1	1
Sam 3	2388	1537	40	5	2	2
Sam 5	2490	1570	49	5	4	2
Sam 6	2081	1362	38	5	3	2
Sam 7	2417	1541	34	0	0	0^5^
Sam 8	2267	1416	53	5	3	1
Sam 9	2284	1354	34	4	1	1
Sam 10	2570	1760	51	5	2	1
Sam 11	2277	1491	44	7	2	1

^1^Sample 4 is not included in this table, as it is composed of pooled DNA from sample 4a and 4b. Slightly different analysis parameters were used for that sample.

^2^NS: nonsynonymous, SS, splice site.

^3^Number of variations remaining after filtering for variations in FA genes only.

^4^pathogenic clues are obtained from *in silico* analysis using Align-GVGD, Polyphen-2, and SIFT [7–9].

^5^This patient is a carrier for a large deletion and a deep intronic mutation. The large deletion is detectable by a separate tool, and the intronic variation is filtered out at this stage.

**Table 3:** Mutation detection in unclassified FA patients.

Sample	Gene	Mut 1	Effect 1	Mut 2	Effect 2	Note
Unc1	BRCA2	c.T8067A	p.C2689X	c.9672_9673insA	p.I3224fs	[10, 11]
Unc2	FANCD2	c.904C>T	p.R302W	c.2715+1G>A	splicing	[12, 13]
Unc3	FANCI	c.G3041A	p.C1014Y	c.G3041A	p.C1014Y	Novel
Unc4	FANCL	c.755_756insAT	p.M252fs	c.755_756insAT	p.M252fs	Novel
Unc5	—	NF*	—	NF*	—	—

Annotations are based on the following transcripts: BRCA2, NM_000059.3; FANCD2, NM_033084.3; FANCI, NM_001113378.1; FANCL, NM_001114636.1.

*NF = not found.
